# Analysis of biological systems using bioimpedance spectroscopy: a critical review of technological convergence and translational challenges

**DOI:** 10.3389/fbioe.2026.1872322

**Published:** 2026-07-14

**Authors:** Ilena Torres-Grau, Francisco Cuadros-Salcedo, Javier Ramos-Maganés, Justo García-Sanz-Calcedo

**Affiliations:** 1 School of Industrial Engineering, University of Extremadura, Badajoz, Spain; 2 Metanogenia, Badajoz, Spain; 3 BIOBEE Microelectronics and BioQ Food Quality, Badajoz, Spain

**Keywords:** bioimpedance spectroscopy, biological systems, biosensors, instrumentation, non-invasive monitoring

## Abstract

Bioimpedance spectroscopy (BIS) has emerged as a versatile, non-invasive technique for real-time electrical characterization of biological systems. By applying alternating current and analyzing the resulting complex impedance, BIS provides insights into cellular structure, tissue properties, and physiological processes, and can also detect pathogenic bacteria. This article presents a scoping review of recent advances in BIS, conducted under the Arksey and O’Malley framework and PRISMA-ScR guidelines, and analyzes 46 studies published between 2015 and 2025 that address BIS applications across biological systems. The strongest evidence was identified in fluid management and hydration monitoring (39.1%), body composition assessment (17.4%), and lymphedema monitoring (15.2%), whereas applications in tissue characterization, cellular systems, agriculture, machine learning-assisted diagnostics, and biosensing technologies remain at lower levels of translational maturity. Recent advances in instrumentation, sensor design, microfluidics, wearable systems, and computational analysis are critically examined. The review identifies major technological barriers, including a lack of standardized acquisition protocols, device-dependent variability, limited interoperability, heterogeneous modeling approaches, and insufficient multicenter validation. Despite challenges related to standardization, modeling of heterogeneous systems, and measurement reproducibility, BIS continues to demonstrate potential across biomedical, biotechnological, and industrial applications. Its ability to provide continuous, real-time, and non-destructive measurements supports its growing use in diagnostic platforms, therapeutic monitoring, and industrial biosensing applications. This review provides an integrated perspective on current developments, limitations, and future directions of BIS-based technologies.

## 1 Introduction

Bioimpedance spectroscopy (BIS) has emerged as a powerful and versatile technique for non-destructive electrical characterization of biological systems (Ciambrone et al., 2004; Noddeland et al., 2024; Stupin, 2018a; Swami et al., 2024; Xu et al., 2016). By applying a low-amplitude alternating current over a wide frequency range and analyzing the resulting complex impedance, BIS provides quantitative information about the structural and functional properties of cells, tissues, and biological fluids. Its non-invasive nature, real-time capability, and label-free operation make it particularly attractive for biomedical research, clinical diagnostics, and biotechnological monitoring (Ben Atitallah et al., 2022; Furst and Francis, 2019; Kassanos, 2021; McGlennen et al., 2023; Montalibet et al., 2023; Noddeland et al., 2024; Novak et al., 2024).

The theoretical foundation of BIS rests on the frequency-dependent electrical behavior of biological matter. Equivalent circuit models—most notably the Cole–Cole model and dispersive RC network representations—are commonly used to interpret impedance spectra in terms of physiologically meaningful parameters (Ben Atitallah et al., 2022; Calvo et al., 2020; Fernandez et al., 2015; Mallick et al., 2023; Nasir and Al Ahmad, 2020; Odry et al., 2021). These models account for extracellular resistance, membrane capacitance, and intracellular conductivity, which vary with tissue composition and physiological state. In addition to conventional outputs related to fluid distribution, Cole-derived parameters, such as cell membrane capacitance (Cm), have recently attracted growing attention as potential biomarkers of cellular integrity and function, extending the scope of BIS toward the assessment of tissue microstructure and cellular health (Brantlov et al., 2025; Matthie, 2008). At low frequencies, electrical current predominantly flows through the extracellular compartment, whereas at higher frequencies it penetrates cell membranes, thereby providing information about intracellular structures and membrane integrity. This frequency-dependent behavior enables BIS to probe biological systems at multiple structural scales (Abasi et al., 2022; Kassanos, 2021; Search et al., 2024).

Distinct dispersion regions have been identified in the impedance spectrum. The α-dispersion (approximately 1–10 kHz) is generally associated with ionic diffusion in the extracellular environment. The β-dispersion (approximately 10 kHz–10 MHz) is primarily linked to cell membrane polarization and is widely used to assess membrane integrity, hydration status, and tissue composition. At higher frequencies, the δ- and γ-dispersions are related to intracellular macromolecules and the dielectric properties of water. Therefore, selecting appropriate frequency windows is critical and strongly dependent on the biological target and measurement configuration (Abasi et al., 2022; Caytak et al., 2019; Kassanos, 2021; Odry et al., 2021; Tang et al., 2020).

Among the wide range of bioimpedance techniques, the main ones include BIS, single-frequency bioimpedance analysis (SF-BIA), multi-frequency bioimpedance analysis (MF-BIA), and electrical impedance spectroscopy (EIS). Although they all share the same underlying electrical principles, they differ in the number of frequencies used, the mathematical model, and their intended applications. SF-BIA uses a single excitation frequency, typically 50 kHz, and is widely used for rapid assessment of body composition or hydration. However, it offers limited discrimination between extracellular and intracellular water compartments and relies heavily on population-specific equations (Earthman et al., 2007; Haverkort et al., 2015). MF-BIA extends the analysis to multiple discrete frequencies, allowing estimation of fat mass, fat-free mass, and appendicular muscle mass in older adults (Gába et al., 2015; Thomson et al., 2007; Yamada et al., 2013). BIS goes a step further by fitting the entire impedance spectrum to equivalent circuit models to estimate extracellular and intracellular fluid volumes with less reliance on regression equations (Earthman et al., 2007; Jaffrin and Morel, 2008). In contrast, EIS is a broader term for measuring frequency-dependent impedance, applicable not only to biological systems but also to biosensing and materials characterization; therefore, BIS can be considered a biological application of EIS focused on living systems (Bera, 2014; Radogna, 2023). Moreover, BIS provides access to spectral parameters, such as phase angle and model-derived electrical properties, that may offer additional information on cellular integrity, hydration status, and tissue structure beyond conventional single-frequency measurements (Ward and Brantlov, 2023). In this review, BIS is treated as the central analytical framework, while SF-BIA studies are included only when they provide a clinically relevant reference point for assessing hydration or body composition, and MF-BIA studies are considered when they help contextualize the added value of spectral measurements relative to BIS.

Owing to this multi-scale sensitivity, BIS has been applied across a broad range of biological contexts. At the cellular level, impedance-based approaches enable real-time monitoring of cell growth, adhesion, differentiation, and viability (Odry et al., 2021; Sebastian et al., 2021; Stupin et al., 2021; Xu et al., 2016). In clinical medicine, BIS contributes to body composition analysis and fluid balance assessment (Huang et al., 2013; Noddeland et al., 2024; Prasad and Roy, 2020; Sunny et al., 2019; Tara and Islam, 2024), dialysis monitoring (Baghbani et al., 2025; Costa et al., 2022), cardiovascular evaluation (Accardi et al., 2021; Hong et al., 2019; Ng et al., 2024; Ritu et al., 2024; Urzeniczok et al., 2023), lymphedema detection, and oncological applications (Braun et al., 2017; Burinaru et al., 2022; Gonzalez-Landaeta & Antonio González-Díaz, 2023; Kamal et al., 2023; Stupin et al., 2021; Xu et al., 2016; Zhang et al., 2025). Beyond human health, the technique has also been implemented in plant physiology, food quality control, microbiological detection, and bioprocess monitoring (Elamine et al., 2024; Feng et al., 2021; Guermazi et al., 2014; Ibba et al., 2020; Kluza et al., 2025; Kovács et al., 2025; Lin et al., 2012; Lin et al., 2018; Riaz et al., 2023; Watanabe et al., 2021; Xu et al., 2016).

Recent advances in sensor miniaturization, flexible and wearable electrodes, microfluidic integration, and embedded electronics have further broadened its applicability. In parallel, integrating advanced signal processing and machine learning techniques has enhanced the interpretability of impedance spectra, enabling improved tissue classification, physiological state prediction, and pattern recognition in complex biological datasets (Burinaru et al., 2022; Fernandez et al., 2015; Kassanos, 2021; Noddeland et al., 2024; Pavlin et al., 2021; Sebastian et al., 2021; Stupin, 2018b; Xu et al., 2016). These developments have expanded the potential of BIS beyond conventional measurement applications, supporting its use as a multiparametric sensing platform for diagnostic and monitoring research.

Despite the rapid expansion of the field, significant challenges remain. Existing literature reveals considerable heterogeneity in electrode configurations, frequency selection strategies, modeling approaches, and calibration procedures. This methodological variability complicates cross-study comparison and limits standardization, particularly in clinical translation (Delano et al., 2022; Fernandez et al., 2015; Stupin et al., 2021). Furthermore, interpreting impedance spectra in heterogeneous biological systems remains complex, requiring robust modeling frameworks and improved reproducibility across devices and experimental setups.

Although numerous reviews have addressed specific applications of bioimpedance, such as body composition analysis, cancer diagnostics, and cell-based impedance sensing, a comprehensive, integrative analysis of recent technological advances in BIS across biological systems remains scarce. In particular, there is a need to critically synthesize developments in instrumentation, sensor architectures, frequency-dependent methodologies, and computational analysis within a unified framework.

Previous reviews have predominantly focused on individual application domains, including body composition assessment, cancer diagnostics, lymphedema monitoring, and cell-based impedance sensing. In contrast, relatively little attention has been paid to comparing BIS applications across biological scales and to identifying common technological and translational barriers shared across these domains. Moreover, few reviews have assessed technology readiness and translational maturity alongside methodological and instrumentation-related developments.

Consequently, this review offers a structured, critical overview of advances in bioimpedance spectroscopy for biological systems from 2015 to 2025. Beyond compiling reported applications, the review emphasizes technological convergence, methodological limitations, barriers to standardization, device interoperability, modeling challenges, and readiness for translation. Particular attention is given to identifying the factors that currently hinder the transition of BIS from proof-of-concept and research settings to clinical, industrial, and biotechnology applications. This interdisciplinary approach is intentional; rather than treating each area of application as an isolated topic, the study uses them as representative cases to identify technological limitations, methodological constraints, and transfer barriers that recur across different biological systems.

## 2 Materials and methods

### 2.1 Review design and methodology

**FIGURE 1:**
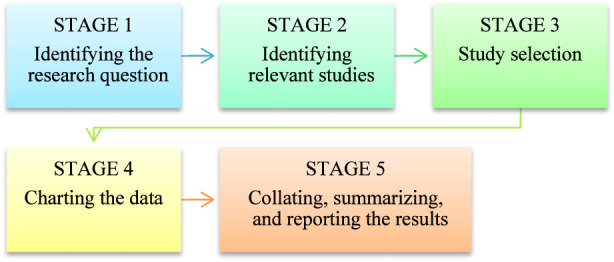
Methodological framework for scoping reviews according to Arksey and O’Malley, 2005.

The present study was structured as a scoping review, a methodology widely used to map key concepts within a field of research and to synthesize available evidence without restricting itself to a specific study type or outcome. The methodological framework proposed by (Arksey and O’Malley, 2005), which is organized into five clear stages, is presented in Figure 1.

To strengthen the quality and transparency of the review, Arksey and O’Malley’s framework was complemented by the updated PRISMA-ScR guidelines (Preferred Reporting Items for Systematic Reviews and Meta-Analyses extension for Scoping Reviews), which were developed to improve the methodological quality and transparency of scoping reviews. PRISMA-ScR provides a 22-item checklist that addresses all steps in the review process, including clearly stated objectives, detailed sources of information, study selection methods, search strategies, and data synthesis approaches (Tricco et al., 2018). Reporting followed the PRISMA-ScR guidelines, and the completed checklist is provided in Supplementary Table S1.

The decision to conduct a scoping review was based on the growing interest in, and diversity of, recent applications of bioimpedance spectroscopy (BIS) in biological systems, including human tissues, cells, and clinical monitoring, and on the need to consolidate this knowledge within the field of biomedical engineering.

For this review, we defined BIS as a technique that measures the complex electrical impedance of a biological system in response to an alternating current applied across a broad frequency range. This measurement uncovers the electrical properties of the medium, such as resistance (the real part), which relates to tissue conductivity, and reactance (the imaginary part), which relates to the polarization capacity of cell membranes and internal dielectric structures. These parameters enable the inference of various structural and physiological properties of the system under study (Abasi et al., 2022; Calvo et al., 2020; Kanoun et al., 2022; Tara and Islam, 2024).

### 2.2 Research questions

The following research questions guided the review.

- What are the main biological applications of BIS reported between 2015 and 2025?
- Which frequency ranges and measurement configurations are most commonly employed?
- What technological advances in instrumentation and sensor design have been introduced?
- What methodological limitations and standardization challenges persist?

### 2.3 Eligibility criteria

To ensure the relevance and quality of the studies included in this review, predefined inclusion and exclusion criteria were applied, as summarized in Table 1.

**TABLE 1:** Inclusion and exclusion criteria were considered in this scoping review.

Inclusion criteria	Exclusion criteria
Publications between 2015 and 2025	Studies without full-text access
Articles published in peer-reviewed scientific journals	Studies on bioimpedance spectroscopy not involving biomedical applications
Studies analyzing applications of bioimpedance spectroscopy in human tissues, cells, or clinically relevant biological samples	Opinion papers, essays without a defined methodology, or non-systematic narrative reviews
Documents available in English or Spanish	Review articles

### 2.4 Search strategy and information sources

The bibliographic search was conducted in the Scopus and Web of Science (WoS) databases because of their broad coverage and indexing of high-quality biomedical journals, as well as their advanced filtering capabilities for year, document type, subject area, and language.

The search employed controlled terms and keywords combined using Boolean operators. In the advanced search, the strategy was applied to title, abstract, and keywords. The search string used was:

“Bioimpedance Spectroscopy” AND (“Biological Systems” OR cells OR tissue OR clinical OR humans OR bacteria OR plant).

The search strategy focused on “bioimpedance spectroscopy” because this review aimed to analyze frequency-dependent spectral approaches and their translational challenges. Although related terms such as BIA and EIS are common in the literature, these methods were not used as primary search terms to preserve conceptual specificity and maintain a clear focus on BIS. Studies were included if they used multifrequency bioimpedance devices and if impedance spectra or BIS-derived parameters were central to the methodology or interpretation, thereby enabling comparison of established clinical applications with new spectral approaches.

Additionally, manual searches were conducted using the reference lists of key articles (snowballing) to identify relevant literature that might not have been retrieved by automated database tools. The searches were conducted in December 2025 using the advanced search functions in Scopus and Web of Science. The complete search string, databases consulted, search fields, and filters were predefined before screening to improve reproducibility. Backward citation tracking was used only as a supplementary strategy and may have preferentially identified highly cited or closely connected publications; therefore, it was not used as a primary source for study identification. This review aims to identify representative studies that illustrate the main biological applications, technological advances, and translational challenges of BIS.

The complete database search strings and filtering procedures are provided in the Supplementary Data: Full search strategy.

### 2.5 Study selection

The article selection process was conducted in three sequential stages.

1. Automatic filtering by publication year, document type, and full-text availability.
2. Manual review of titles and abstracts to exclude studies that did not meet the eligibility criteria.
3. Full-text review to confirm methodological and thematic relevance.

All stages were conducted by a single reviewer following a predefined protocol based on the Arksey and O'Malley framework and PRISMA-ScR recommendations. While this approach ensured methodological consistency throughout screening, eligibility assessment, and data extraction, it may have increased the risk of selection bias compared with independent dual-reviewer screening.

Because this review was designed as a scoping review to identify representative studies across multiple biological application domains, the screening process prioritized thematic relevance, methodological contribution, and translational significance over an exhaustive quantitative synthesis. Reasons for exclusion included non-biological applications; studies focused exclusively on conventional BIA or non-biological EIS; insufficient methodological detail; lack of full-text availability; and limited relevance to the review’s objectives.

### 2.6 Data extraction and analysis

A data extraction table was created to organize the following information for each selected article.

- Author and year of publication
- Type of study (experimental, clinical, review, technical)
- Main objective of the study
- Frequency range used in impedance measurements
- Type of biological system analyzed (cells, tissues, humans, plants, *etc.*)
- Measurement setup and method (conventional BIS, multi-electrode, portable, *etc.*)
- Key results, applications, and technical findings
- Technology Readiness Level (TRL), when sufficient information was available to estimate the maturity of the proposed technology

Technology Readiness Levels (TRLs) were assigned descriptively based on the maturity framework proposed by the European Commission (Héder, 2017). The classification was estimated from the information provided in each study, considering the stage of technological development, the validation environment, and the degree of implementation. Studies limited to laboratory validation or proof-of-concept demonstrations were generally classified within TRLs 3–5, whereas technologies evaluated in clinical settings or nearing routine implementation were classified within TRLs 6–9. Therefore, the assigned TRLs should be interpreted as approximate indicators of translational maturity rather than as formal regulatory assessments.

Because this was an exploratory review, no formal risk-of-bias assessment was conducted, nor was the methodological quality of the studies formally rated. Instead, the evidence was characterized descriptively by study design, sample size, technology readiness level (TRL), and the consistency of findings across application domains, enabling a narrative appraisal of the relative strength and maturity of the available evidence.

The extracted data were analyzed qualitatively and grouped into major application domains (e.g., clinical diagnostics, physiological monitoring, agriculture, and bioprocesses). In addition, a descriptive quantitative analysis was conducted, focusing on frequency ranges, sensor configurations, study objectives, and translational maturity. Summary figures and comparative tables were generated to identify current trends and challenges in applying BIS to biological systems.

## 3 Results

### 3.1 Study selection

The bibliographic search was carried out in December 2025 using the Scopus and WoS databases, applying the previously defined search strategy. In Scopus, a total of 1.320 records were initially retrieved. After applying the time filter (2015–2025), this number was reduced to 934 documents, of which 725 were scientific articles. After restricting the search to English- and Spanish-language articles, 709 potentially relevant articles were selected.

In Web of Science, the initial search yielded 1.701 records. After filtering by publication year, 1.210 documents were retained, including 921 articles. After applying the language filter, 912 articles were considered eligible.

The combination of both databases resulted in 1.621 articles. For duplicate detection, the references were exported in. ris format and processed with the Mendeley bibliographic manager, enabling more accurate deduplication. This procedure identified 574 duplicate records, resulting in a total of 1,047 unique articles.

The remaining articles were screened for relevance to the research questions and eligibility criteria. Study selection was conducted in two stages: first, titles and abstracts were screened for eligibility; second, full texts of potentially relevant studies were reviewed. Articles were excluded for reasons including unavailability of full texts, applications outside biological systems, redundant or repetitive studies, low relevance or out-of-scope status, and other methodological issues. As expected in a broad scoping review, most excluded records were studies outside the intended scope, including non-biological applications of impedance spectroscopy, studies focused on conventional BIA without spectral BIS analysis, materials characterization studies using EIS, and publications lacking sufficient methodological detail to enable meaningful comparison. The selection process was guided by the objective of identifying representative studies that illustrate the main biological applications, technological developments, and translational challenges of BIS.

**FIGURE 2:**
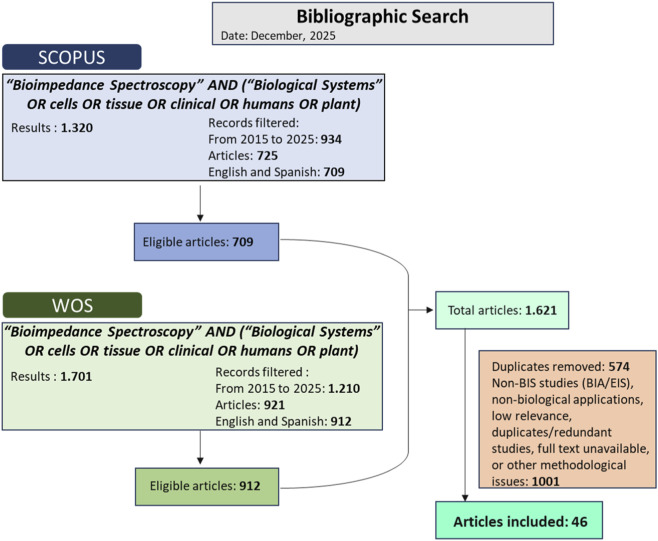
PRISMA flow diagram for article selection process.

Following this process, 46 studies met all inclusion criteria and were included in this scoping review. The complete process of identification, selection, and exclusion is summarized in the PRISMA flow diagram (Figure 2) in accordance with PRISMA-ScR guidelines.

**FIGURE 3:**
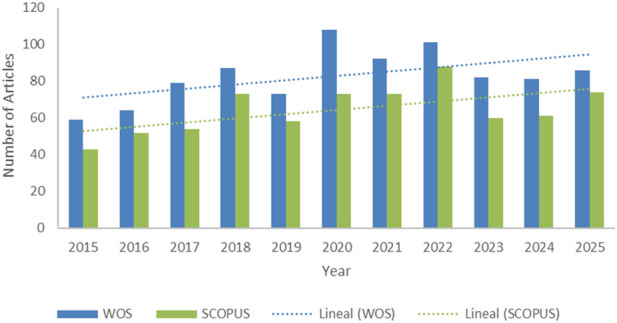
Annual number of articles related to bioimpedance spectroscopy retrieved from Web of Science and Scopus databases between 2015 and 2025 according to the search strategy used in this study.

To contextualize the evolution of research activity in BIS, a temporal analysis was conducted of the number of publications retrieved from WoS and Scopus using the search strategy described above. The annual distribution of publications between 2015 and 2025 is shown in Figure 3.

Overall, the results show sustained scientific activity in this field over the last decade, with a gradual increase in publications and a notable peak around 2020–2022. Although there have been small fluctuations in recent years, the general trend indicates that BIS remains an active and evolving area of research.

### 3.2 Data extraction and synthesis

Key information from each selected study was extracted and organized into a summary table. Table 2 presents the main evaluated parameters: author and year, study type, objective, frequency range used, biological application, measurement method, key findings, and TRL. To provide an additional level of characterization for the studies, each study was assigned a descriptive technology readiness level (TRL) based on its experimental, preclinical, clinical, or implementation maturity, enabling a comparison of the technological and translational development of BIS applications across biological domains.

**TABLE 2:** Summary of bioimpedance spectroscopy applications in biological systems.

Ref.	Study type	Main objective	Frequency range	Biological system	Sample size	Measurement setup	Electrode configuration and material	ML/AI used	Key findings	TRL*
Lin et al. (2018)	Prospective cohort	Body composition and mortality in CKD	5–1,000 kHz	CKD patients	326 participants	BCM	Not specified (integrated within commercial device)	No	High LTI predicted lower mortality, superior to BMI	8-9
Kugananthan et al. (2017)	Cohort	Maternal adiposity and breast milk composition	4–1,000 kHz	Lactating women	283 milk samples from 59 women	SFB7 tetra-polar bioelectrical impedance analyzer	Not specified (integrated within commercial device)	No	Higher fat mass linked to leptin/protein levels	7-8
Ng et al. (2022)	Prospective observational	Fluid status impact on body composition in PD	5 kHz–1 MHz	PD patients	101 participants	BCM	Not specified (integrated within commercial device)	No	LTI/FTI not biased by volume status	7-8
Noddeland et al. (2024)	Experimental	Wearable sensor for fluid balance	1 kHz–1 MHz	Healthy adults	27 participants	Re:Balans® patch	Not specified (integrated within commercial device)	No	Detected induced hypovolemia; ↑ extracellular resistance	5-6
Sanchez-Perez et al. (2022)	Clinical validation	Pulmonary fluid monitoring	5–150 kHz	HF patients	10 healthy +14 HF patients	Wearable multimodal BIS	Tetrapolar configuration using standard 3 M electrodes	No	Resistance ratio tracked edema reduction	6-7
Hecking et al. (2018)	Retrospective cohort	Overhydration and mortality in HD	Multifrequency	HD patients	38.614 participants	BCM	Not specified (integrated within commercial device)	No	FO strongly predicted mortality	8-9
van Ruiten et al. (2022)	RCT analysis	Drug effects on fluid volume	Multifrequency	T2D patients	66 participants	ImpediMed BIS	Not specified (integrated within commercial device)	No	Dapagliflozin reduced extracellular volume	8-9
Hayes et al. (2017)	Prospective cohort	Lower limb lymphedema	Extrapolated R0	Gynecologic cancer	408 participants	BIS limb ratio	Not specified	No	Early lymphedema detection	8-9
Kittiskulnam et al. (2017)	Cohort	Sarcopenia vs mortality in HD	4–1,000 kHz	HD patients	645 participants	SFB7	Not specified (integrated within commercial device)	No	Physical performance > muscle mass as a predictor	7-8
Bettenfeld et al. (2023)	Experimental (LOC)	Single-cell BIS monitoring	1 kHz–100 MHz	THP-1 cells	Not specified	Microfluidic LOC	10 pairs of Pt microelectrodes	No	Real-time electro permeabilization monitoring	3-4
Haerteis et al. (2018)	Clinical/experimental	PKLK and volume retention	5–1,000 kHz	CKD patients, mice	171 participants	BCM + electrophysiology	Not specified (integrated within commercial device)	No	Potential role of PKLK correlated with overhydration	7-8
Ehlayel et al. (2023)	Pilot study	Fluid assessment in pediatric HD	5 kHz- 1 MHz	Pediatric HD	8 participants	Xitron Hydra 4,200	Not specified (integrated within commercial device)	No	BIS overestimated TBW vs D2O	7-8
Gridneva et al. (2016)	Experimental clinical	Feeding effect on infant BIS	3–1,000 kHz	Infants	48 participants	SFB7	Not specified (integrated within commercial device)	No	Feeding did not alter resistance significantly	4-5
Halavina et al. (2024)	RCT	BIS-guided decongestion in TAVR	5–1,000 kHz	Aortic stenosis	232 participants	BCM	Not specified (integrated within commercial device)	No	Reduced HF hospitalization and mortality	8-9
Mabrouk et al. (2020)	Experimental/clinical	Ankle edema monitoring	5–100 kHz	Humans	11 participants	Wearable BIS sensor	Tetrapolar configuration, Ag/ AgCl gel-based electrodes	No	Differential score detected edema reliably	4-5
Saadé et al. (2024)	Experimental (in vitro)	Placental barrier monitoring	5 Hz–1 MHz	BeWo cells	Not specified	AD2 + electrodes	Bipolar configuration, Ag/AgCl electrodes	No	TEER changes confirmed monolayer formation	3-4
Schork et al. (2019)	Prospective	SGLT2 inhibitors and fluid changes	5–1,000 kHz	T2D patients	27 participants	BCM	Not specified (integrated within commercial device)	No	Initial fluid loss, long-term fat reduction	8-9
Tinsley et al. (2023)	Validation study	Validation of total body water (TBW) estimation using BIS and SF-BIA against deuterium oxide (D2O)	50 kHz - > SF-BIA and multifrequency (BIS, 256 frequencies)	Human subjects	130 participants	SF-BIA (Quantum V) and BIS (SFB7)	Tetrapolar configuration, Ag/ AgCl electrodes for BIS and 8 electrodes for SF-BIA	No	BIS showed no significant differences vs D2O and higher accuracy; SF-BIA underestimated TBW; both methods showed low error	7-8
Dekker et al. (2016)	Prospective cohort	Fluid overload + inflammation	Multifrequency	HD patients	8.883 participants	BCM	Tetrapolar configuration, Ag/ AgCl electrodes	No	Combined FO + inflammation ↑ mortality	8-9
Schwaiger et al. (2020)	Pilot intervention	Empagliflozin post-transplant	Standard BCM	Renal transplant	14 participants	BCM	Tetrapolar configuration, Ag/ AgCl electrodes	No	Reduced extracellular fluid transiently	7-8
Li et al. (2023)	Experimental	Multisine BIS method	2–997 kHz	RC phantom models simulating human whole-body impedance	Not specified	FPGA + ADC + DAC	Not specified	No	Reduced sampling cost; ∼1% error	3-5
Mussnig et al. (2024)	Comparative	Device/electrode errors	4.88–1,000 kHz	Healthy volunteers	40 participants	BCM vs Cella	Tetrapolar configuration, Ag/ AgCl electrodes, and gold-plated copper electrodes	No	Devices not interchangeable	4-5
Tara and Islam (2024)	Clinical + ML	Cellular state classification	5–1,000 kHz	Students	1.000 participants	Bodystat 5,000	Not specified (integrated within commercial device)	Sí	RF achieved 100% classification accuracy	3-5
Hung et al. (2015)	Cohort + animal	Volume overload in CKD	5–1,000 kHz	CKD + rats	338 humans and 40 rats	BCM + ImpediVet	Not specified (integrated within commercial device)	No	FO predicted renal/CV events	8-9
Schneider et al. (2017)	Clinical study	Skin sodium and LVH	5–1,000 kHz	CKD patients	99 participants	BCM +23Na-MRI	Not specified (integrated within commercial device)	No	Skin sodium linked to LV mass	8-9
Kim et al. (2019)	Experimental	Organic electrodes for plants	100 Hz–1 MHz	Plants	Leaves from 14 plant species	Printed polymer electrodes	Bipolar configuration, polymeric electrodes (PProDOT-Cl)	No	Long-term, non-invasive monitoring	4-5
Antlanger et al. (2017)	Prospective cohort	HF prevalence in HD	5 kHz–1 MHz	HD patients	105 participants	BCM	Not specified (integrated within commercial device)	No	70% met HF criteria	8-9
Baghbani et al. (2021)	In vitro	Lung nodule detection	50 kHz–5 MHz	Human lung tissue	286 lung tissue samples obtained from 38 participants	Custom probe	Tetrapolar configuration, spherical electrodes	Sí	>95% classification accuracy	4-5
Dylke et al. (2016)	Diagnostic validation	BIS thresholds for lymphedema	Standard BIS	Breast cancer	68 participants	SFB7	Not specified (integrated within commercial device)	No	High sensitivity/specificity	8-9
Eyre et al. (2022)	Cross-sectional	Malnutrition diagnosis	5 kHz–1 MHz	CKD5 patients	90 participants	BCM vs DXA	Not specified (integrated within commercial device)	No	Poor sensitivity for GLIM criteria	6-7
Kilbreath et al. (2016)	Prospective	Lymphedema risk factors	Not specified	Breast cancer	540 participants	BIS serial monitoring	Not specified	No	Early detection predictive	8-9
Pinheiro-Castro et al. (2024)	Clinical technical	Improved infant modelling	5–1,000 kHz	Infants	72 participants	Bodystat 5,000	Tetrapolar configuration, AgCl gel electrodes (Bodystat 0,515)	No	Improved FFM prediction	4-5
Search et al. (2024)	Experimental + ML	Tissue identification	1–200 kHz	Porcine tissues	598 Ex vivo + in vivo porcine tissues	Custom AD5940 probe	Tetrapolar configuration, gold-coated beryllium copper electrodes	Sí	70% NN classification accuracy	3-5
Shah et al. (2024)	RCT	Early lymphedema prevention	Not specified	Breast cancer	919 participants	L-Dex U400	Not specified (integrated within commercial BIS device)	No	92% risk reduction	8-9
Visser et al. (2025)	Prospective	FFM vs CT comparison	5–1,000 kHz	CKD patients	60 participants	BCM	Not specified (integrated within commercial BIS device)	Sí	Good group correlation	7-8
Kit-Chung Ng et al. (2018)	Prospective	Asymptomatic FO in PD	5–1,000 kHz	PD patients	311 participants	BCM	Not specified (integrated within commercial BIS device)	No	FO predicted mortality	7-8
Boyages et al. (2015)	Prospective	Liposuction in lymphedema	Not specified (commercial device)	Advanced lymphedema	104 participants	ImpediMed L-Dex	Not specified (integrated within commercial BIS device)	No	89% limb volume reduction	8-9
Feng et al. (2021)	Experimental/ agricultural validation	Impedance analyzer (IM3570, Hioki)	1 kHz–4 MHz	Rice seed	100 seeds total	IM3570	Bipolar configuration, copper electrodes with a diameter of 1 mm	Sí	Impedance correlated with moisture distribution and microstructural degradation	4-5
Feldman et al. (2015)	Clinical report	LYMPHA prevention	Not specified (commercial device)	Breast cancer	40 participants	L-Dex	Not specified (integrated within commercial BIS device)	No	Reduced lymphedema incidence	7-8
Kilgore et al. (2018)	Retrospective	Early detection with BIS	Not specified	Breast cancer	146 participants	Serial BIS	Not specified	No	82% resolved with early intervention	8-9
Pichonnaz et al. (2015)	Validation study	Post-TKA swelling	BIS (R0 extrapolated)	TKA patients	25 participants	SFB7	Not specified (integrated within commercial BIS device)	No	Higher sensitivity than tape	7-8
Wang et al. (2020)	Experimental	Non-invasive deep-brain stimulation	≈1–2 kHz	Murine brain	10 mice	custom stimulator with integrated bioimpedance measurement	6 electrodes (2 cranial, 2 cheek, 2 grounding)	No	Real-time BIS improves stimulation stability and targeting	3-4
Zoccali et al. (2017)	Epidemiological cohort	Chronic FO and mortality	Standard BCM	HD patients	39.566 participants	BCM	Not specified (integrated within commercial BIS device)	No	FO exposure strongly predictive	8-9
Sunny et al. (2019)	Experimental	Skin hydration sensor	1–50 kHz	Human skin + phantoms	Not specified	Low-cost tetrapolar sensor	Tetrapolar configuration, Ag/AgCl gel electrodes	No	<3% deviation; glucose monitoring potential	4-5
Kovács et al. (2025)	Experimental	Design of the BIS device for plants and water stress detection	1 Hz–100 kHz	Plants	230 impedance spectra/samples	BIS with gold electrodes + PSO algorithm	Tetrapolar configuration, gold electrodes with a diameter of 1 mm	Sí	Significant correlation with water stress	4-5
Novak et al. (2024)	Experimental (prototype validation, in vitro)	Non-invasive diagnosis of limb compartment syndrome using bioimpedance	1–100 kHz	Chicken, duck, piglet limbs	10 measurements	Custom BACS device (dual-channel, AD5933)	Bipolar configuration, Kendall ECG electrodes (H92SG and CA610)	No	Strong correlation with increased intramuscular pressure	3-4

Abbreviations are defined in the Nomenclature section.

* The authors have estimated the TRL, values based on the European Commission’s maturity framework; therefore, they should be interpreted as indicative rather than definitive classifications.

To provide a more comprehensive critical synthesis, the included studies were grouped by primary area of application, translational maturity, and strength of evidence. Table 3 summarizes the distribution of the studies, the main biological systems investigated, the typical technology readiness level (TRL), and the key strengths and limitations identified in each area of application. Studies were assigned to a single primary application domain based on their main objective, and categories were treated as mutually exclusive for descriptive quantitative synthesis.

**TABLE 3:** Critical comparison of BIS application domains.

Application domain	Studies (%)	Predominant biological system	Typical TRL*	Main strengths	Main limitations	Overall evidence
Fluid management and hydration monitoring	18 (39.1%)	Dialysis, Chronic Kidney Disease (CKD), heart failure patients	7–9	Strong clinical validation, prognostic value, intervention-guided management	Device non-interchangeability, population-specific models, limited standardization	High
Body composition assessment	8 (17.4%)	Adults, infants, dialysis patients	7–8	Non-invasive assessment of fat mass, lean mass and body water	Dependence on predictive equations, hydration assumptions, population-specific calibration	High
Lymphedema monitoring	7 (15.2%)	Breast and gynecological cancer patients	8–9	Early detection, preventive intervention, strong clinical outcomes	Variable diagnostic thresholds, device differences, limited protocol harmonization	High
Tissue characterization and oncology	3 (6.5%)	Human and animal tissues	3–5	High discrimination capability and diagnostic potential	Small cohorts, proof-of-concept studies, limited external validation	Moderate-Low
Cellular and microfluidic systems	2 (4.3%)	Cell cultures and organ-on-chip models	3–4	High sensitivity to cellular morphology, viability and barrier integrity	Limited reproducibility, dependence on electrode geometry and modelling assumptions	Low
Plant and agricultural applications	3 (6.5%)	Seeds and plants	4–5	Non-destructive physiological monitoring and stress assessment	Environmental variability, heterogeneous tissues, limited field validation	Low
Machine learning-assisted applications	2 (4.3%)	Multiple biological systems	3–5	Enhanced classification and pattern recognition capabilities	Small datasets, overfitting risk, lack of external validation and limited clinical utility evidence	Low
Instrumentation and sensing technologies	3 (6.5%)	Experimental platforms, sensors and phantoms	3-5	Improved acquisition speed, portability and measurement capabilities	Limited biological validation and translational evidence	Low

* TRL, values were estimated qualitatively by the authors based on the reported validation environment and degree of technological implementation.

The quantitative distribution of studies by area of application showed a strong concentration of available evidence in fluid management and hydration monitoring (18/46; 39.1%), followed by body composition assessment (8/46; 17.4%) and lymphedema monitoring (7/46; 15.2%). Together, these clinically oriented applications accounted for 33 of the 46 included studies (71.7%) and were generally associated with the highest levels of technology readiness (TRL 7–9) and the strongest evidence base. In contrast, tissue characterization and oncology (3/46; 6.5%), plant and agricultural applications (3/46; 6.5%), cellular and microfluidic systems (2/46; 4.3%), and machine learning-assisted applications (2/46; 4.3%) were represented by fewer studies and were predominantly classified as technologies with a low-to-intermediate TRL. These findings indicate that, although BIS has expanded into a wide range of biological domains, the strongest translational evidence remains concentrated in fluid monitoring, body composition assessment, and lymphedema treatment, while most emerging applications are still based primarily on proof-of-concept and pilot-scale research.

In addition to the application-domain analysis, quantitative information on sample size, electrode configuration, and materials, as well as the use of machine learning approaches, was extracted from the included studies and incorporated into Table 2. This additional characterization provides a broader overview of methodological trends and technological development across BIS applications.

## 4 Discussion

**FIGURE 4:**
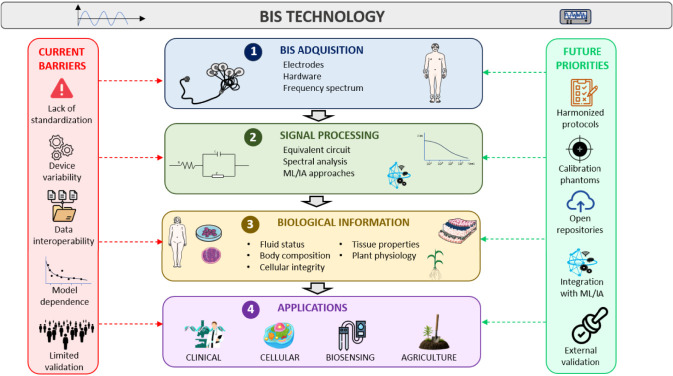
Translational framework of bioimpedance spectroscopy (BIS) across biological systems.

Bioimpedance spectroscopy (BIS) has evolved from a biophysical technique focused on a single area into a cross-cutting tool with applications spanning clinical medicine, cell engineering, microbiology, agronomy, and emerging biotechnological systems. The reviewed studies suggest considerable versatility and demonstrate BIS’s potential to provide real-time, non-invasive, marker-free information across a wide range of biological conditions. Figure 4 provides an integrated overview of the main technological components, biological applications, translational bottlenecks, and future priorities identified throughout this review. However, the main barrier to broader translational adoption of BIS is not sensitivity, but the lack of spectral, geometric, and modeling standardization across biological scales (Hecking et al., 2018; Kugananthan et al., 2017; Ng et al., 2022; Noddeland et al., 2024; Sanchez-Perez et al., 2022; van Ruiten et al., 2022). Until this fragmentation is addressed, BIS data will remain only partially interoperable between devices, laboratories, and biological domains.

### 4.1 Clinical evidence and translational maturity

In clinical physiology, BIS appears to have its strongest evidence in fluid management, body composition assessment, and lymphedema monitoring. Studies in peritoneal dialysis and hemodialysis show that extracellular water quantification predicts mortality and cardiovascular events, outperforming traditional indicators such as body mass index (BMI) or isolated blood pressure (Dekker et al., 2016; Hecking et al., 2018; Hung et al., 2015; Kit-Chung Ng et al., 2018; Lin et al., 2018; Visser et al., 2025; Zoccali et al., 2017). Multifrequency devices such as the Body Composition Monitor (BCM) and ImpediMed SFB7 BIS device have enabled the separation of lean mass, fat mass, and overhydration (Kugananthan et al., 2017; Lin et al., 2018; Ng et al., 2022). Randomized controlled trials further suggest that BIS-guided intervention may improve outcomes in selected settings, including heart failure and lymphedema (Boyages et al., 2015; Feldman et al., 2015; Halavina et al., 2024; Shah et al., 2024). Additional validation studies have supported the accuracy of BIS-derived total body water estimates compared with deuterium oxide dilution, although population-specific physiological factors continue to influence accuracy (Tinsley et al., 2023). Nevertheless, important methodological limitations remain. Most predictive equations are population-specific and show reduced generalizability across age groups and clinical conditions (Brantlov et al., 2016; Ehlayel et al., 2023; Eyre et al., 2022; Visser et al., 2025). Pediatric validations reveal a systematic overestimation of total body water relative to isotopic dilution (Ehlayel et al., 2023; Eyre et al., 2022), and clinical outcomes remain supported by relatively few multicenter trials focused on hard endpoints. In hemodialysis, BIS-derived muscle mass correlates with mortality, although physical performance may be a stronger predictor than lean mass (Kittiskulnam et al., 2017), and fluid overload remains highly prevalent in this population (Antlanger et al., 2017). As a result, the prognostic value of BIS is strong, but its causal integration into standardized therapeutic algorithms remains incomplete outside nephrology and lymphedema.

Lymphedema monitoring illustrates both the potential and current limitations of translational BIS. Prospective studies show that L-Dex-based monitoring detects subclinical extracellular fluid accumulation earlier than limb volumetric measurements and reduces progression when early intervention is triggered (Boyages et al., 2015; Dylke et al., 2016; Feldman et al., 2015; Hayes et al., 2017; Kilbreath et al., 2016; Kilgore et al., 2018; Shah et al., 2024). However, the diversity of threshold criteria (1 standard deviation, 2 standard deviations, or fixed L-Dex values), combined with hemodynamic fluctuations in the early postoperative period and non-interchangeability between devices, leads to variability in diagnostic interpretation (Dylke et al., 2016; Feldman et al., 2015; Kilgore et al., 2018; Mussnig et al., 2024). Device non-interchangeability adds another layer of variability, and experimental evidence shows that electrode geometry and reuse can alter resistance measurements enough to affect clinical interpretation (Mussnig et al., 2024). The limitation is therefore structural, as calibration standards and algorithmic transparency remain insufficient to ensure multicenter reproducibility.

Beyond volume quantification, BIS can detect dynamic changes in composition. Studies evaluating SGLT2 inhibitors describe an initial contraction in extracellular volume followed by a sustained reduction in fat mass, demonstrating the technique’s ability to differentiate between transient fluid shifts and structural tissue changes (Schork et al., 2019; Schwaiger et al., 2020; van Ruiten et al., 2022). Likewise, correlations between extracellular expansion, epithelial Sodium Channel (ENaC) activation, and tissue sodium accumulation associated with ventricular hypertrophy suggest that impedance-derived markers reflect integrated physiological states, rather than being limited to simple volumetric metrics (Haerteis et al., 2018; Schneider et al., 2017). Even so, these associations remain inferential because BIS quantifies electrical indicators associated with membrane capacitance and ionic conductivity, rather than direct molecular mechanisms. The lack of systematic integration with biochemical biomarkers or imaging techniques limits mechanistic understanding.

### 4.2 Emerging applications beyond clinical monitoring

At the cellular and microfluidic level, BIS shows high sensitivity to morphological changes, cell adhesion, and barrier integrity. These observations are consistent with previous analyses of cellular dielectric behavior and impedance-based cell characterization models (Nasir and Al Ahmad, 2020). Xu et al. (2016) and Bettenfeld et al. (2023) validated the detection of cell-substrate impedance and individual cells over the range of 1 kHz–100 MHz using lab-on-a-chip (LOC) devices with platinum microelectrodes and hydrodynamic traps. Saadé et al. (2024) developed a system to monitor syncytialization and tight junction integrity in BeWo b30 cells (a placental barrier model) using measurable resistances and capacitances over the frequency range of 5 Hz to 1 MHz. These approaches offer high temporal resolution and mechanistic specificity, although the results remain sensitive to electrode placement, microchannel geometry, and Cole model adjustments, limiting quantitative reproducibility (Pinheiro-Castro et al., 2024; Stupin et al., 2021).

The emerging literature on oncology, tissue characterization, machine learning-assisted diagnostics, and prototype-based biosensing systems illustrates the growing expansion of BIS beyond conventional body composition assessment. Several proof-of-concept studies report classification accuracies above 95% for lung nodules, tissue discrimination, and cellular state identification (Baghbani et al., 2021; Gonzalez-Landaeta & Antonio González-Díaz, 2023; Search et al., 2024; Tara and Islam, 2024), while prototype systems have demonstrated sensitivity to clinically relevant physiological changes, including compartment syndrome and bioimpedance-guided neurostimulation (Novak et al., 2024; Wang et al., 2020). Despite these promising results, most studies rely on small datasets, limited external validation, and heterogeneous acquisition protocols spanning frequency ranges from hundreds of hertz to hundreds of megahertz (Baghbani et al., 2021; Search et al., 2024; Tara and Islam, 2024). As shown in Table 2, machine learning applications were generally evaluated on relatively limited datasets and under study-specific electrode configurations, which may further restrict model transferability across devices and measurement settings. Consequently, the risk of overfitting remains substantial, particularly when model development and testing are performed within the same dataset (Tara et al., 2025). In addition, many studies provide limited information regarding model interpretability and feature relevance, making it difficult to determine whether classification decisions are driven by biologically meaningful impedance characteristics or by dataset-specific patterns. Moreover, high classification accuracy does not necessarily translate into clinical utility, as evidence regarding patient outcomes, decision-making impact, and cost-effectiveness remains scarce (Abasi et al., 2022). Challenges also remain regarding integration into routine clinical workflows, including interoperability with existing diagnostic systems, regulatory requirements, and prospective validation in real-world clinical settings. Combined with the intrinsic variability of bioimpedance signals across individuals and clinical states, these limitations suggest that current BIS-based machine learning studies should still be considered proof-of-concept rather than ready-to-implement clinical solutions. Future studies should therefore prioritize external validation and multicenter evaluation.

#### 4.2.1 Plant and agricultural applications

`Outside the field of medicine, BIS is also finding applications in plant systems and precision agriculture. Feng et al. (2021) showed that certain impedance and phase characteristics allow for highly accurate differentiation between fresh and aged rice seeds, correlating spectral changes with moisture distribution and microstructural degradation (Feng et al., 2021). Lin et al. (2018) reported a significant association between impedance signals and phosphorus status in tomato plants, whereas Watanabe et al. (2021) proposed a novel LTO-based indicator to monitor low-temperature storage effects in sweet potato tissues (Lin et al., 2018; Watanabe et al., 2021). Although promising, field applications remain limited by environmental variability, tissue heterogeneity, and the lack of standardized acquisition protocols.

### 4.3 Modeling and interpretation challenges

Classical spectral modeling remains fundamental to BIS interpretation. The Cole model remains the dominant framework because its parameters (R_0_, R ∞ , Cm, and α) have direct biophysical meaning linked to extracellular and intracellular conduction pathways (Abasi et al., 2022; Aggas et al., 2023; Mohamed et al., 2025; Mussnig et al., 2024). In clinical contexts, extrapolation of R_0_ and R ∞ allows estimation of extracellular and intracellular water behaviors using mixing theories such as the Hanai model, providing physiologically interpretable metrics that data-only algorithms cannot directly provide (Mohamed et al., 2025; Mulasi et al., 2015). Beyond conventional body composition and fluid parameters, BIS is increasingly being explored to derive additional biomarkers from the Cole model, particularly cell membrane capacitance (C_m_), which may reflect membrane integrity, cellular structure, and tissue health. However, estimation of C_m_ remains highly dependent on model assumptions, measurement quality, frequency range, and electrode configuration (Abasi et al., 2022; Buendia et al., 2011; Matthie, 2008; Yamada et al., 2013). Recent studies also propose C_m_ as a potential biomarker of cellular health and function. For example, changes in C_m_ have been associated with disease status and altered cell membrane integrity in pediatric nephrotic syndrome, supporting its potential physiological relevance beyond conventional fluid measurements (Brantlov et al., 2019). However, significant limitations remain regarding standardization, interpretability, measurement quality, and clinical validation (Brantlov et al., 2025; Brantlov et al., 2017b; Garr Barry et al., 2023). Therefore, although C_m_ extends the scope of BIS beyond volume estimation to cellular-level characterization, its translational use remains in its infancy and requires harmonized protocols and larger validation cohorts before routine clinical implementation.

### 4.4 Technological advances and standardization barriers

Advances in engineering have rapidly expanded BIS’s technical capabilities. Multisine excitation implemented with Field-Programmable Gate Arrays (FPGAs) enables multiple frequencies to be generated and processed simultaneously, reducing sampling requirements without compromising spectral resolution. In addition, wearable tetrapolar sensors enable continuous monitoring, and printed organic electrodes facilitate low-cost, adaptable integration into biological interfaces (Kim et al., 2019; Li et al., 2023; Noddeland et al., 2024; Sunny et al., 2019). These innovations have improved the technical capabilities of BIS systems across several application domains. However, hardware-related limitations remain relevant in many emerging applications, particularly where device interoperability, calibration procedures, and measurement reproducibility have not yet been standardized.

Methodological variability—including excitation protocols, electrode configuration, and device-specific signal processing—continues to limit reproducibility and interoperability across platforms. In addition, the lack of standardized procedures for quantifying measurement uncertainty further complicates the interpretation and comparison of impedance-derived parameters across devices and populations. Previous guidelines and reviews have emphasized the importance of establishing standardized acquisition procedures, quality control protocols, and validated models specific to each population, to ensure the reliability of estimates derived from impedance (Brantlov, et al., 2017a; Kyle et al., 2004a; 2004b; Ward, 2019). Furthermore, impedance measurements are influenced by multiple preanalytical variables, including hydration status, body position, skin preparation, and electrode placement, which can introduce variability and hinder comparability across studies and populations (Brantlov, et al., 2017b). Added to this are device-dependent factors, as equipment based on similar bioimpedance principles can yield markedly different impedance values and body composition estimates, thereby limiting interoperability and direct comparison of results across platforms (Bennett et al., 2024; Mussnig et al., 2024; Silva et al., 2019). Future advances will therefore depend not only on improvements in sensors and signal processing but also on harmonized calibration strategies, uniform reporting standards, and robust validation frameworks that ensure reproducible measurements across devices, laboratories, and clinical settings.

### 4.5 Future directions

**FIGURE 5:**
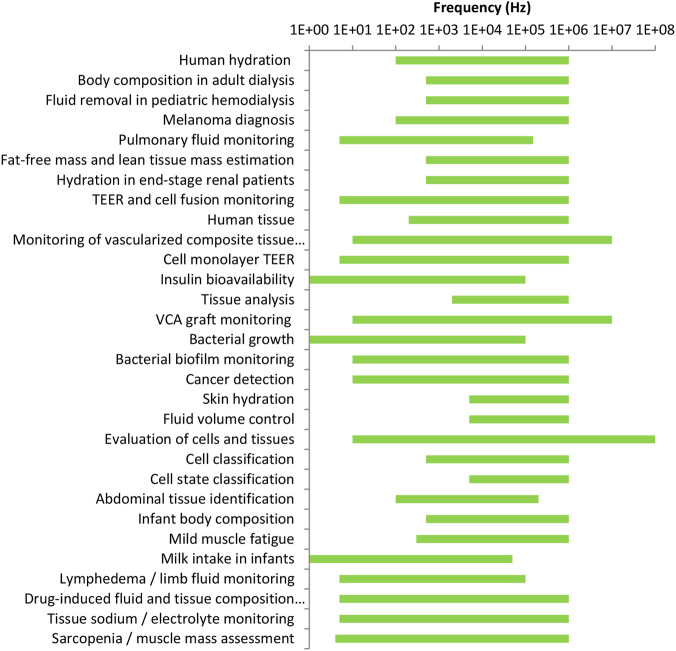
Frequency ranges used in different BIS applications.

The frequency distribution summarized in Figure 5 illustrates the substantial variability in frequency selection across BIS applications, reflecting differences in physiological targets and measurement objectives.

Overall, BIS shows consistent performance in detecting macroscopic physiological changes, particularly extracellular volume expansion, but loses accuracy when applied to fine tissue discrimination or individualized compositional quantification. This limitation does not stem from physical deficiencies in the technique, but rather from modeling assumptions about tissue homogeneity, isotropy, and constant resistivity that are rarely met *in vivo*. Muscle anisotropy, dynamic redistribution of electrolytes, and impedance at the electrode-skin interface introduce systematic uncertainties that are rarely addressed in clinical studies.

This quantitative analysis, further summarized in Table 3, confirms a marked imbalance in translational maturity across BIS applications. Clinical domains such as fluid management, body composition assessment, and lymphedema monitoring are supported by relatively mature evidence, whereas cellular systems, plant applications, tissue characterization, machine learning-assisted diagnostics, and biosensing platforms remain largely supported by proof-of-concept and early validation studies.

Several efforts are still required to facilitate broader translational adoption of BIS, including multicenter validation studies, standardized spectral repositories, and integration with complementary biomarkers and imaging modalities to support more mechanistically grounded biological interpretation.

### 4.6 Limitations of the review

This study presents considerations for interpreting its results and, in turn, guides future lines of research. First, the broad scope adopted to analyze bioimpedance spectroscopy (BIS) across various biological systems prioritizes identifying common patterns of technological, methodological, and translational nature; therefore, a more specific and detailed analysis of each application area could be developed in subsequent studies dedicated to specific fields. In this regard, some emerging areas could benefit from more in-depth and specialized evaluations. Second, the search strategy focused primarily on BIS and related spectral approaches. Although it was reinforced by manual review of relevant references, future reviews could expand the search terms—including “bioimpedance analysis” (BIA) or “electrical impedance spectroscopy” (EIS)—to achieve even more comprehensive coverage of the available literature. Finally, the use of a single reviewer for study selection and data extraction may have introduced selection and interpretation bias. Future reviews should incorporate independent dual-reviewer screening and consensus procedures to improve methodological robustness.

## 5 Conclusion

Bioimpedance spectroscopy (BIS) has become a widely used non-invasive technique for fluid monitoring and longitudinal assessment of body composition, with applications in multiple biological systems ranging from clinical physiology to cellular bioengineering and microbiology. Recent studies confirm its ability to provide real-time, marker-free data, and the integration of portable sensors and miniaturized systems has substantially expanded its reach into clinical, pediatric, and experimental settings.

However, the translational maturity of BIS remains uneven. While the strongest evidence is concentrated in fluid management, body composition, and lymphedema monitoring, many emerging applications are still supported mainly by proof-of-concept or early validation studies. Across domains, the main barriers to broader implementation are not related to signal sensitivity but to heterogeneity in acquisition protocols, device-dependent variability, reliance on model assumptions, and limited multicenter validation.

To further expand the translational impact of BIS, it is essential to promote reproducible standards for acquisition and analysis, develop universal calibration tools, and encourage integration with biomarkers, imaging modalities, and computational strategies. Continued methodological harmonization and validation efforts will be critical to support broader implementation across different application domains.

## Nomenclature

AD2: Analog Discovery 2 (measurement device)
ADC: Analog-to-digital converter
AI: Artificial Intelligence
BACS: Bioimpedance Analyzer for Compartment Syndrome
BIS: Bioimpedance Spectroscopy
BIA: Bioimpedance Analysis
BCM: Body Composition Monitor
BMI: Body Mass Index
CKD: Chronic Kidney Disease
Cm: Cell membrane capacitance
CT: Computed Tomography
CV: Cardiovascular
D2O: Deuterium Oxide
DAC: digital-to-analog converter
DXA: Dual-Energy X-ray Absorptiometry
ECG: Electrocardiogram
EIS: Electrical Impedance Spectroscopy
ENaC: Epithelial Sodium Channel
FFM: Fat-Free Mass
FPGA: field-programmable gate array
FO: Fluid Overload
FTI: Fat Tissue Index
GLIM: Global Leadership Initiative on Malnutrition
HF: Heart Failure
HD: Hemodialysis
LOC: Lab-on-a-Chip
LTO: Low-temperature storage indicator
LTI: Lean Tissue Index
LV: Left Ventricle
LVH: Left Ventricular Hypertrophy
MF-BIA: Multi-Frequency Bioimpedance Analysis
ML: Machine Learning
MRI: Magnetic Resonance Imaging
NN: Neural Network
PD: Peritoneal Dialysis
PKLK: Plasma Kallikrein
PRISMA-ScR: Preferred Reporting Items for Systematic Reviews and Meta-Analyses Extension for Scoping Reviews
PSO: Particle Swarm Optimization
PProDOT-Cl: poly(3,4-propylenedioxythiophene chloride)
RC: Resistor–Capacitor
RCT: Randomized Controlled Trial
**R**_**0**_: Resistance at zero frequency
**R** ∞: Resistance at infinite frequency
RF: Random Forest
SFB7: ImpediMed SFB7 BIS device
SF-BIA: Single-Frequency Bioimpedance Analysis
SGLT2: Sodium–Glucose Cotransporter 2
T2D: Type 2 Diabetes
TAVR: Transcatheter Aortic Valve Replacement
TEER: Transepithelial Electrical Resistance
TBW: Total Body Water
TKA: Total Knee Arthroplasty
TRL: Technology Readiness Level
WoS: Web of Science
α: Alpha dispersion (low-frequency region)
β: Beta dispersion (mid-frequency region)
γ: Gamma dispersion (high-frequency region)
Δ: Delta dispersion (associated with intracellular macromolecules)
