# Supplementary material for: Analysis of biological systems using bioimpedance spectroscopy: a critical review of technological convergence and translational challenges
Source: Front Bioeng Biotechnol. 2026 Jul 14;14:1872322. doi: 10.3389/fbioe.2026.1872322 (PMC13408212; doi:10.3389/fbioe.2026.1872322)
Supplement: Supplementary file 1 [file Table1.docx]

Supplementary Material

# Supplementary Table S1. PRISMA-ScR Checklist

Supplementary Table 1. PRISMA-ScR checklist indicating where each reporting item is addressed in the manuscript.

| Section | Item | PRISMA-ScR Checklist Item | Location in manuscript |
| --- | --- | --- | --- |
| Title | 1 | Identify the report as a scoping review | Title |
| Abstract | 2 | Structured summary | Abstract |
| Introduction | 3 | Rationale | Introduction |
| Introduction | 4 | Objectives | Section 2.2 Research Questions |
| Methods | 5 | Protocol and registration | No formal review protocol was registered. |
| Methods | 6 | Eligibility criteria | Section 2.3 Eligibility Criteria; Table 1 |
| Methods | 7 | Information sources | Section 2.4 Search Strategy and Information Sources |
| Methods | 8 | Search | Section 2.4 Search Strategy and Information Sources |
| Methods | 9 | Selection of sources of evidence | Section 2.5 Study Selection |
| Methods | 10 | Data charting process | Section 2.6 Data Extraction and Analysis |
| Methods | 11 | Data items | Section 2.6 Data Extraction and Analysis |
| Methods | 12 | Critical appraisal of individual sources of evidence | Not performed. As this was a scoping review, no formal critical appraisal or risk-of-bias assessment was conducted; see Section 2.6 Data Extraction and Analysis. |
| Methods | 13 | Summary measures | Not applicable for scoping reviews |
| Methods | 14 | Synthesis of results | Section 2.6 Data Extraction and Analysis |
| Methods | 15 | Risk of bias across studies | Not applicable for scoping reviews |
| Methods | 16 | Additional analyses | Not applicable for scoping reviews |
| Results | 17 | Selection of sources of evidence | Section 3.1 Study Selection; Figure 2 |
| Results | 18 | Characteristics of sources of evidence | Table 2 |
| Results | 19 | Critical appraisal within sources of evidence | Not performed. Consistent with the exploratory nature of this scoping review, no formal critical appraisal or risk-of-bias assessment of individual studies was conducted. |
| Results | 20 | Results of individual sources of evidence | Table 2 |
| Results | 21 | Synthesis of results | Section 3.2 Data Extraction and Synthesis; Table 3 |
| Results | 22 | Risk of bias across studies | Not applicable for scoping reviews |
| Results | 23 | Additional analyses | Not applicable for scoping reviews |
| Discussion | 24 | Summary of evidence | Discussion |
| Discussion | 25 | Limitations | Discussion (final paragraphs) |
| Discussion | 26 | Conclusions | Section 5 Conclusions |
| Funding | 27 | Funding | Funding section |

# Supplementary Data. Full search strategy

The searches were conducted in December 2025 using the advanced search functions available on Scopus and Web of Science (WoS).

## Scopus

Search field: Title, Abstract and Keywords

Search string: "Bioimpedance Spectroscopy" AND ("Biological Systems" OR cells OR tissue OR clinical OR humans OR bacteria OR plant)

Filters applied:

- Publication years: 2015–2025
- Document type: Article
- Languages: English and Spanish

## Web of Science (WoS)

Search field: Topic (Title, Abstract, Author Keywords and Keywords Plus)

Search string: "Bioimpedance Spectroscopy" AND ("Biological Systems" OR cells OR tissue OR clinical OR humans OR bacteria OR plant)

Filters applied:

- Publication years: 2015–2025
- Document type: Article
- Languages: English and Spanish

## Additional search strategy

Backward citation tracking (snowballing) was conducted using the reference lists of selected key articles to identify potentially relevant studies not retrieved by the database searches. This supplementary strategy was used solely to support study identification and not as a primary source for selecting evidence. The search strategy, eligibility criteria, and screening process were predefined prior to study selection to improve transparency and reproducibility.
